# Cancer organoid applications to investigate chemotherapy resistance

**DOI:** 10.3389/fmolb.2022.1067207

**Published:** 2022-12-13

**Authors:** Kenji Harada, Naoya Sakamoto

**Affiliations:** ^1^ Graduate School of Biomedical and Health Sciences, Hiroshima University, Hiroshima, Japan; ^2^ Division of Pathology, Exploratory Oncology Research and Clinical Trial Center, National Cancer Center, Kashiwa, Japan

**Keywords:** cancer, organoid, drug resistance, drug-tolerant persisters, drug screening, precision medicine

## Abstract

In clinical practice, a large proportion of cancer patients receive chemotherapy, yet tumors persist or acquire resistance; removing this obstacle could help to lower the number of cancer-related fatalities. All areas of cancer research are increasingly using organoid technology, a culture technique that simulates the *in vivo* environment *in vitro*, especially in the quickly developing fields of anticancer drug resistance, drug-tolerant persisters, and drug screening. This review provides an overview of organoid technology, the use of organoids in the field of anticancer drug resistance research, their relevance to clinical information and clinical trials, and approaches to automation and high throughput.

## 1 Introduction

**FIGURE 1:**
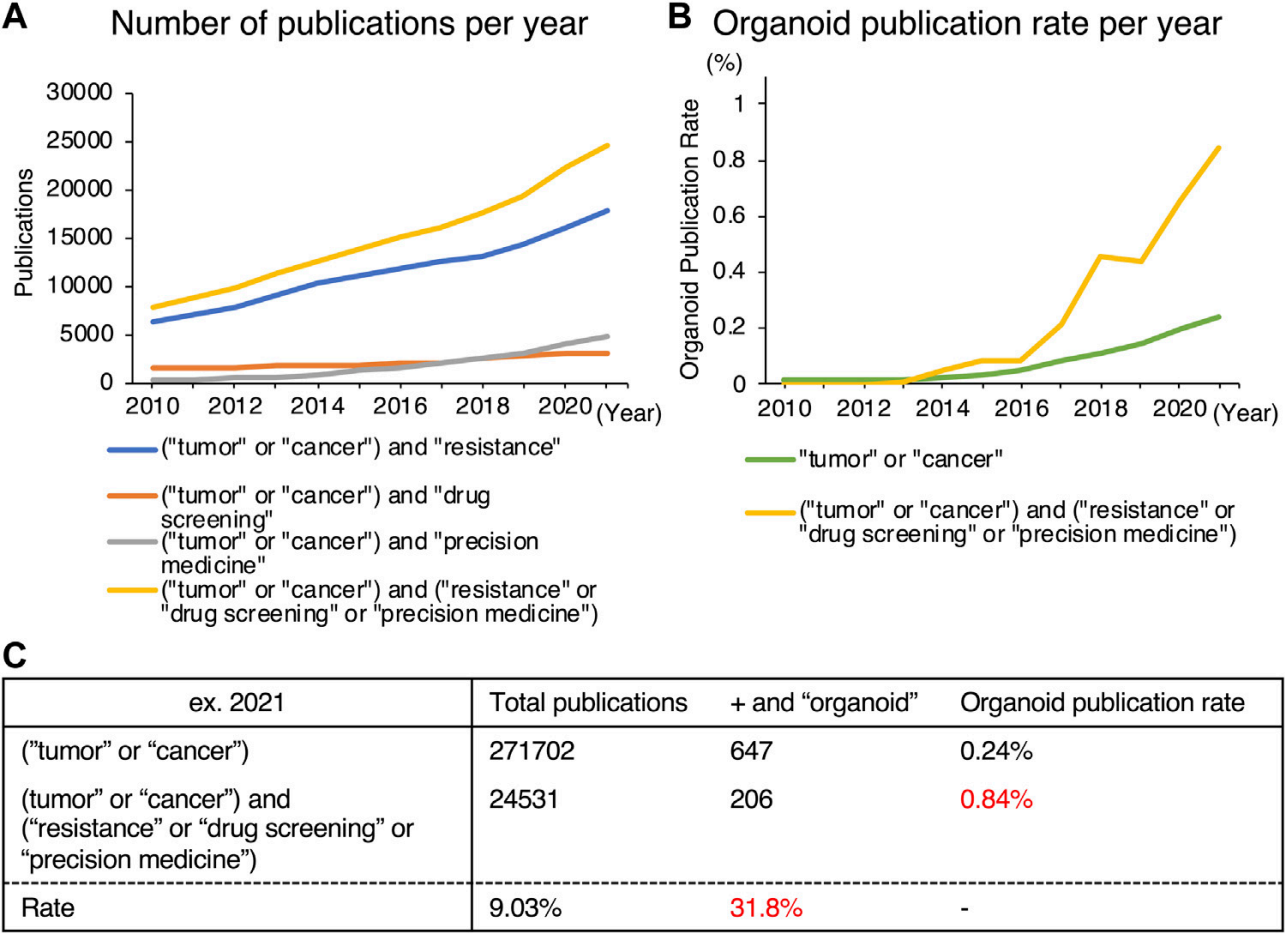
Trends in publications on organoids obtained from a PubMed search. **(A)** Number of publications in specific areas [resistance (blue), drug screening (orange), precision medicine (grey), or all (yellow)] in cancer-related articles. **(B)** Proportion of organoid-related publications in cancer research (green) and in anticancer drug-related areas (yellow). **(C)** Detailed data of **(B)**.

In 2020, 10 million individuals died from cancer according to CA 2021 (Sung et al., 2021), indicating the necessity of additional research into remedies. Chemotherapy is currently administered either alone or in conjunction with radiation or surgical resection. Even though some patients respond well to chemotherapy, tumors frequently become resistant to it during treatment. For this reason, studies on anticancer drug resistance have been stepped up regardless of the type of cancer (Mikubo et al., 2021; Lohan-Codeço et al., 2022; Otaegi-Ugartemendia et al., 2022). Furthermore, it is now well known that tumors are highly heterogeneous and complex, prompting investigations toward personalized medicine aimed at resolving these issues so that optimal chemotherapy can be selected for each patient (Van den Bossche et al., 2022). Studies on the development of novel pharmaceuticals, such as small-molecule chemicals and nucleic acid medicines for the creation of novel therapies, are also gaining momentum (Castro et al., 2021; Mahajan et al., 2021). The number of published references for the terms (“tumor” or “cancer”) and (“resistance” or “drug screening” or “precision medicine”) is rising each year, reaching 24,531 in 2021, according to a PubMed search for those terms (Figure 1A).

In this area of research, although it is essential to look toward quick clinical applications in personalized medicine and/or new drug development, it is also necessary to conduct fundamental research on the mechanisms of anticancer drug resistance and/or the mechanism of action of new drugs, requiring a technology that possesses both characteristics. Cell culture is typically one of the most essential non-clinical models for fundamental studies in cancer research and therapeutic development. Two-dimensional cell line culture has long been a central part of *in vitro* assay (Breslin and O’Driscoll, 2013). Although two-dimensional culture may be handled easily and is inexpensive, it has the disadvantage of being unable to capture the complexity and three-dimensional (3D) structures seen in the human body (Costa et al., 2016). In addition, it was recently discovered that there are significant differences between the gene expression patterns of cell lines and those of *in vivo* models, making the use of cell lines as a research tool a major obstacle to studies aiming for clinical application (Ravi et al., 2015). Moreover, although many candidate molecules for novel anticancer drugs that passed Phase I studies to determine their safety for clinical use move on to Phase II/III studies to examine pharmacological responses, the success rate is low at 13.4%, and numerous attempts to develop novel drugs have failed (DiMasi et al., 2013). The necessity for new preclinical models is suggested to address the high failure rate in clinical trials. Despite being an efficient method, *in vivo* mouse studies are not appropriate for mass screening or the creation of tailored therapy due to high experimental expenses. We should use non-animal preclinical models as well from the standpoint of animal welfare. Various 3D culture techniques have been created as ways of bridging the gap between cell lines and *in vivo* models to address this problem.

**FIGURE 2:**
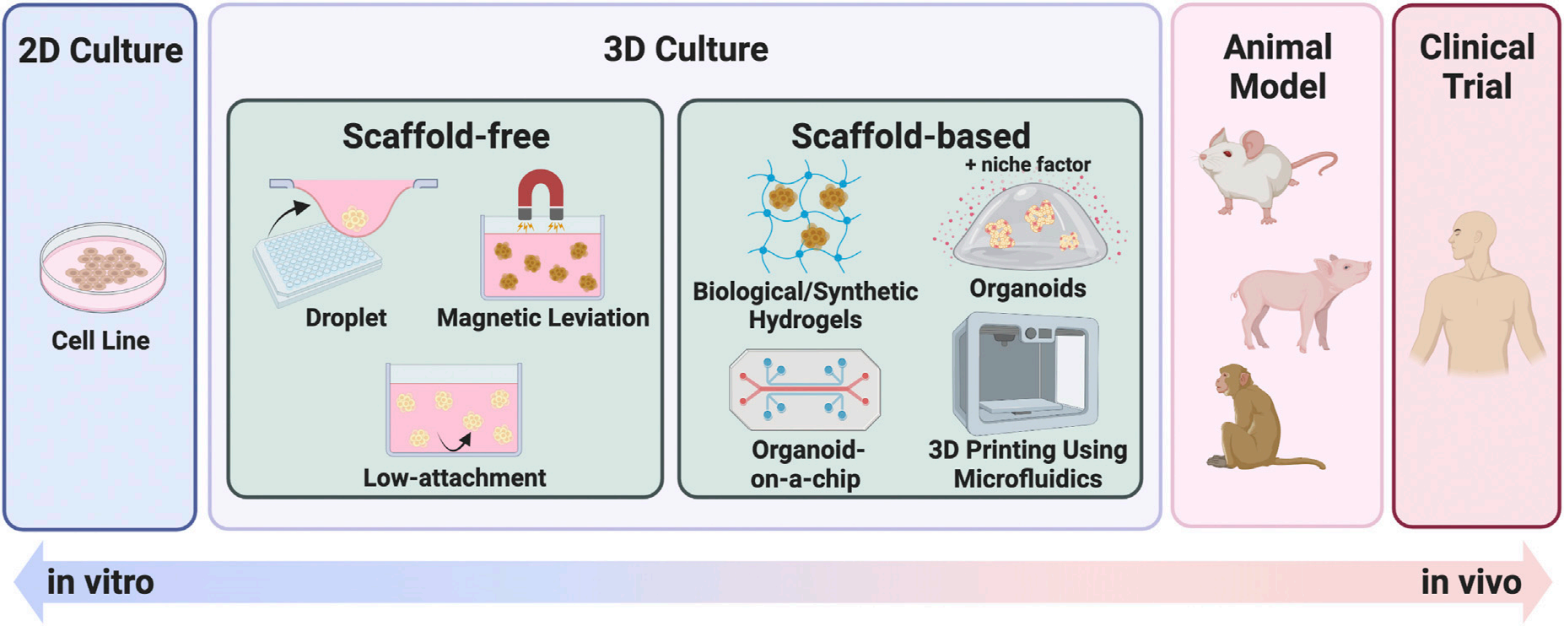
Summary of 3D culture methods. 3D culture methods bridging the gap between 2D culture and *in vivo* models. Each illustration is a scheme of representative 3D culture methods, categorized as scaffold-free or scaffold-based.

While 2D culture is a single layer of cells, 3D culture technology can harbor cells in multiple layers, creating an environment, that is, more similar to that of *in vivo* conditions. The two main categories of 3D culturing methods are scaffold-free and scaffold-based. Figure 2 summarizes these representative culture methods. Scaffold-free technology includes the use of hanging drop microplates, magnetic levitation, and spheroid culture using ultra-low-adhesion coating plates (Kelm et al., 2003; Souza et al., 2010; Vinci et al., 2012). Hanging drop microplates enable the culture of cells in a single drop of liquid medium (Kelm et al., 2003). Magnetic levitation is a technology that uses magnetic nanoparticles injected into the cells and an external magnet to levitate the cell mass in the liquid (Souza et al., 2010). Scaffold-based technologies include natural or engineered polymers and hydrogels (Langhans, 2018), 3D printed scaffolds created by electrospinning (Park et al., 2008), and organoid, a tissue culture technology that uses Matrigel and a niche factor to mimic a microenvironment, leading to self-assembly and self-renewal of cells (Sato et al., 2009). While most of the 3D culture technologies utilize cells derived only from conventional cell lines, the organoid method enables the establishment and maintenance of culture from stem cells derived from primary specimens. The organoids referred to in this review are mainly those derived from primary tumors, not from cell lines. Each of these methods has benefits and drawbacks, and they should be chosen based on the goal of the experiment. Organoids have been widely used in 3D culture techniques in the specific disciplines of anticancer drug resistance, personalized medicine, and novel drug development, which are the subjects of this review. The difference between the percentage of organoid-related literature in these specific areas and that of overall cancer research has diverged significantly (Figure 1B). Furthermore, 206 of the 647 organoid-based cancer articles published in 2021 belonged to these fields, accounting for 31.8% of the total (Figure 1C). One reason for the popularity of organoids is the remarkable rate of establishment derived from primary cancer. This technology enables the detailed characterization of cancer cells for individual patients. Also, organoids can be cultured in a state with a high stem cell content due to their physiological properties, making them useful for studying cancer stem cells (CSCs). Organoids are still at the development stage, but they are being used as a more vivo-like culture method that can replace cell lines as an outstanding technology in this field.

In this review, we first summarize the history of the application of organoids to cancer research, established culture methods, and their similarity to tumor tissues. We then categorize organoids-based anticancer drug-related research into three groups: a study on resistance mechanisms, personalized medicine, and automated high-throughput drug screening methodology.

## 2 Organoids as a pre-clinical model

### 2.1 Organoid history and application

The first investigation into organoids was conducted in 1975 by James G. Rheinwald and Howard Green. They showed that co-culturing primary human keratinocytes and 3T3 fibroblasts produced squamous epithelial colonies that resembled the human epidermis, with cell proliferation in the basal layer and keratinization in the upper layer (Rheinwald and Green, 1975). Subsequently, the understanding of extracellular matrix was improved, and mammary gland-derived cells were actually cultured in a laminin-rich 3D matrix, giving rise to the prototypical model of the current organoids (Barcellos-Hoff et al., 1989; Petersen et al., 1992). For a very long time after that, organoid technology remained in the dark, until in 2009, Sato and others (2009) succeeded in producing mouse intestine organoids. They discovered elements such wingless-related integration site (Wnt), epidermal growth factor (EGF), Noggin, and R-spondin1 (Rspo), which may be crucial in stem cell maintenance, based on past studies on the growth requirements of their maintenance. After adding a combination of these factors to the culture medium, they established long-term culture conditions lasting 8 months for crypt-villus organoids generated from Lgr5-positive stem cells.

Two years later, in 2011, human tissue-derived colon cancer organoids were established by optimizing the culture conditions of mouse intestinal organoids (Sato et al., 2011). In the process of establishing these organoids, they screened various hormones, vitamins, growth factors, and small molecule inhibitors, identifying gastrin, nicotinamide, TGF-beta/Smad inhibitor (A83-01), and p38 MAPK inhibitor (SB202190) as elements that contribute to organoid growth. This led to a rapid acceleration of its application to multiple organs that included prostate cancer (Gao et al., 2014), stomach cancer (Bartfeld et al., 2015), pancreatic cancer (Boj et al., 2015), liver cancer (Broutier et al., 2017), bladder cancer (Pauli et al., 2017), breast cancer (Sachs et al., 2018), ovarian cancer (Kopper et al., 2019), and renal cancer (Schutgens et al., 2019). The combination of the niche factors and their concentrations are highly dependent on the microenvironment of each organ of origin, leading to continued research on their application to other organs.

**FIGURE 3:**
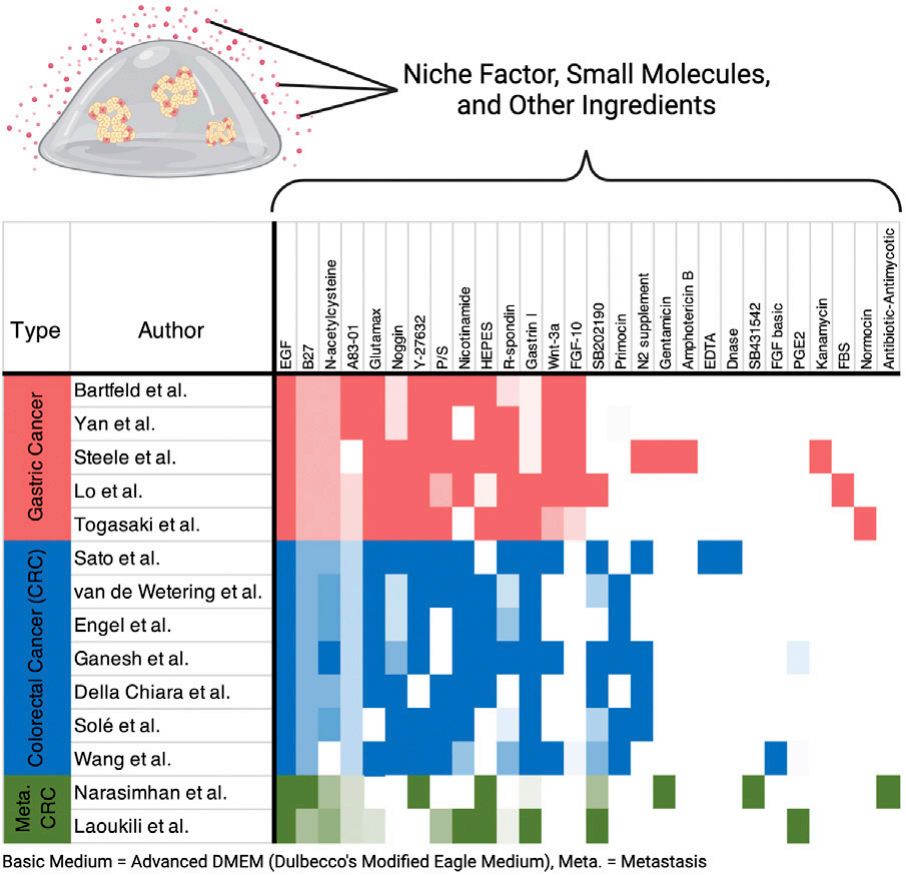
Heatmap of media ingredients. Recipes from representative research papers on gastric cancer and colorectal cancer (CRC) are included in the figure. Color intensity correlates with concentrations of each ingredient. Indescribable concentrations such as conditioned medium were normalized within the ingredients. All the ingredients and concentration details are listed in Supplementary Table S1.

Organoids are now widely used in cancer research, and many researchers have established their own cultures. However, it is being noticed that different compositions of medium are used even for a particular cancer type. For example, a comparison of several papers based on gastric cancer organoids shows that although many additives are common, fibroblast growth factor 10 and gastrin are found in different concentrations, and N2 supplement, A83-01, SB202190, nicotinamide, and fetal bovine serum are either included or absent (Bartfeld et al., 2015; Yan et al., 2018; Steele et al., 2019; Lo et al., 2021; Togasaki et al., 2021) (Figure 3, Supplementary Table S1). Similar variation in niche factors tends to be observed for colorectal cancer (Figure 3, Supplementary Table S1). In addition, it has been reported that small changes in the composition of the culture medium can significantly alter the properties and growth efficiency of the organoids. For example, by removing Wnt and Rspo from the gastric cancer medium, organoids exhibiting the morphology of signet-ring cell carcinoma were reported to become culturable (Togasaki et al., 2021). The use of IGF and FGF2 instead of SB202190 has been reported to improve survival and proliferation rates when performing genome editing of intestinal organoids (Fujii et al., 2018). Considering the above, culture conditions and protocols should be carefully determined taking into account the objectives and methods of the study before starting the experiment.

### 2.2 Biological characteristics of organoids

Although organoids are generally described as a good reproduction of *in vivo* structure, how well organoids recapitulate *in vivo* tissues has always been a subject of discussion. Here, we evaluate the details separately in terms of morphology, genomics, and gene expression.

#### 2.2.1 Histological features

The obvious characteristic of organoids is that their morphological features are similar to those of patient-derived tissues. In the colorectal cancer organoid biobank report by van de Wetering et al. (2015), hematoxylin and eosin (H&E) staining of organoid and patient tissues was compared, which generally showed that cystic or solid features were preserved. Gao et al. (2014) compared tissue, organoids, and xenografts from prostate cancer patients with H&E staining and immunohistochemical stains such as PanCK, and showed that organoids were similar to the *in vivo* samples in most histologic types. Gastric cancer organoids evaluated using H&E staining and immunohistochemical staining for p53 and ERBB2 showed a high degree of tissue and organoid similarity (Bartfeld et al., 2015; Yan et al., 2018). H&E-stained images of pancreatic cancer organoids revealed the formation of organoids similar to the structure of the tissue, consisting of various degrees of dysplastic tall columnar cells resembling low-grade PanINs (Boj et al., 2015). In this study, the immunohistochemical staining images of CAM5.2, p53, and SMAD4 were also shown to be consistent in patient tissues and organoids (Boj et al., 2015). The typical structures of hepatocellular carcinoma, such as the solid structures and pseudoglandular rosettes, were also observed in its organoids. Cholangiocarcinoma organoids also have extensive glandular ductal domains, where cancer cells invade the lumen and grow in a sieve-like structure, as seen in patient tissue (Broutier et al., 2017). Organoids from uterine carcinosarcoma, urothelial carcinoma, and renal cell carcinoma have also shown H&E staining images matching those in patient tissue, organoids, and patient-derived xenografts (PDXs) (Pauli et al., 2017). In a study of breast cancer organoids, immunohistochemical staining for estrogen receptor, progesterone receptor, and HER2 was performed, in addition to H&E staining. It showed that histological subtypes are conserved in the organoids, indicating that the expression pattern of organoids matches that of the patient tissue; however, this expression pattern was not consistent between patient tissues and organoids in all cases, ranging from 70% to 90% concordance in positivity for these target genes (Sachs et al., 2018). H&E staining of ovarian cancer organoids was generally consistent with that of the patient’s tissue, and the expression patterns of paired box gene 8 (PAX8) and p53 were also shown to be consistent (Kopper et al., 2019). A clear comparison of H&E staining of primary tumor and organoids can be found in the main (Gao et al., 2014; Bartfeld et al., 2015; Boj et al., 2015; van de Wetering et al., 2015; Broutier et al., 2017; Pauli et al., 2017; Sachs et al., 2018; Yan et al., 2018) and supplementary figures (Boj et al., 2015; van de Wetering et al., 2015; Broutier et al., 2017) in these references, as well as our previous paper on gastric cancer organoids (Ukai et al., 2020). In summary, the conservation of morphological features has been reported in organoids derived from various organs.

#### 2.2.2 Genomic characteristics

Genomic profiling of organoids from various cancer types has also been shown to be consistent with matched patient-derived tissues. Analysis of 22 colorectal cancer organoid libraries, which included whole exome sequencing, revealed that somatic mutations shared between organoids and biopsy material were on average 88% matched (van de Wetering et al., 2015). Parallel exome sequencing analysis of gastric cancer organoids and frozen tumor tissue from 44 cases confirmed the common presence of most of the major drivers, including TP53, CDH1, and RHOA (Yan et al., 2018). A study of whole exome sequencing of neuroendocrine tumor, pancreatic adenocarcinoma, renal cancer, serous carcinoma of the ovary, urothelial carcinoma, endometrial adenocarcinoma, and leiomyosarcoma showed only minor differences in allele-specific copy numbers and single nucleotide variants between patient tissue and organoids. These minor differences are assumed because organoids do not harbor all subclones and because of the growth progression of organoids (Pauli et al., 2017). Results from whole genome sequencing of breast cancer organoids indicated that copy number alternations in organoids often showed a much cleaner and clearer signal than that in the original patient tissue, which was largely retained even after long-term passaging. For mutations, the majority of cases showed 75%–100% concordance, except in a few cases in which the patient tissue-organoid match was very low (Sachs et al., 2018). The reason for the lack of matching in some cases may be due to intratumoral heterogeneity (Gerlinger et al., 2012). In the analysis of ovarian cancer organoids, the genomic landscape of the organoids, including chromosome and copy number aberrations, was found to closely reflect that of the original tumor. Somatic mutations and amplifications/deletions were also found to be mostly consistent between tumors and organoids (Kopper et al., 2019).

#### 2.2.3 Gene expression at bulk level

Differences in gene expression profiles between the original patient tissues and organoids have been analyzed by microarray, RNA-seq, and related methods. Transcriptome analysis of gastric cancer organoids and corresponding cancer tissues showed a high correlation of expression profiles by histological subtype. However, also found were many organoid-specific down-regulated genes, which were enriched in immune processes, biological adhesion, and extracellular matrix pathways. These findings do not contradict the results that reflect a state of being separated from interactions with the microenvironment *in vivo* (Yan et al., 2018). Similarities were also observed in the RNA-seq results for liver cancer organoids in terms of the correlation of expression patterns between each case and organoid pair and for each subtype (Broutier et al., 2017). Moreover, in a combined study of the results of the expression analysis of organoids in breast cancer and The Cancer Genome Atlas (TCGA) data, they concluded that there was no culture bias from the organoid establishment as the expression patterns of the classification clusters and subtypes observed in TCGA were similar for organoids (Sachs et al., 2018). In glioblastoma organoids, as a comparison of the entire transcriptomes of organoids and corresponding tumor tissues, they indicated that high similarity is maintained over time. However, Jacob et al. (2020) characterized the differentially expressed genes as decreased expression of blood- and immune-related genes yet reported that no organoid-specific genes were up-regulated. The findings for non-small cell lung cancer organoids also suggest that organoid tumor cells can retain most of the key molecular characteristics of their tissue of origin (Shi et al., 2020). RNA-seq of patient-derived colorectal cancer organoids showed that 84% of gene expression matched between tumors and organoids, indicating that colorectal cancer-specific signatures are enriched in organoids (Della Chiara et al., 2021). Organoids from a rare disease, neuroendocrine prostate cancer, have also been shown to have transcriptomes and epigenomes consistent with clinical samples (Puca et al., 2018). Bruun et al. (2020) showed through principal component analysis of gene expression patterns that the correlation coefficient between matched liver metastatic colorectal cancer organoids and tumors was significantly higher than the average between unmatched patient-derived organoids and tumors. In contrast, they also reported that principal component 2 of the tumor samples correlated with an enrichment score calculated based on liver-specific genes, which may reflect the influence of non-malignant cell infiltration in the tumor microenvironment. This could explain the divergence in expression patterns between some tumors and their corresponding organoids (Bruun et al., 2020). Recently, Raghavan et al. (2021) compared RNA-seq and organoid signature analysis of pancreatic cancer tissues that revealed a bias toward established organoids. Pancreatic cancer is classified into classical and basal types, but they found that pancreatic cancer organoids established successfully in long-term culture were biased to the classical type. Overall, the gene expression patterns of organoids are largely consistent with those of the original clinical samples, but the possibility of altered expression patterns depending on culture conditions and their selection pressure, or contamination of non-tumor cells, must be considered.

#### 2.2.4 Gene expression at single-cell level

Recently, transcriptome analysis at the single-cell level has become popular, and now single-cell RNA-seq (scRNA-seq) is also being performed in the field of cancer organoids. Results of scRNA-seq of organoids established from glioblastoma and its tissues further support that organoids recapitulate the heterogeneity and molecular properties found in the corresponding parent tumors (Jacob et al., 2020). Wang et al. (2022) performed paired scRNA-seq of tissue and colorectal cancer organoids established using conditioned or chemical-defined media. They reported that organoids maintained the characteristics of biological tissues, and the conditioned medium was superior for long-term culture in terms of genomic, epigenomic, and transcriptomic features. Meanwhile, scRNA-seq analysis of pancreatic cancer organoids and their biopsy material showed that the expression signatures are highly distinct, and the emergence of expression patterns present only in organoids has also been described (Raghavan et al., 2021). The number of scRNA-seq studies comparing *in vivo* tissue and organoids is currently insufficient, and as shown in the two reports presented here, the conclusions are still controversial, and further investigation is required.

## 3 Anti-cancer drug resistance mechanism

Tumor recurrence after chemotherapy remains an issue not fully resolved; hence, the use of organoids to overcome this problem is being actively investigated. One reason for the wide use of cancer tissue-derived organoids in this field is that cancer organoids can be cultured in a state rich in CSCs, which are thought to play an important role in the acquisition of chemotherapy resistance (Fujii and Sato, 2017). In addition to CSCs, several studies have recently proposed the importance of “drug-tolerant persister cells” or “dormant cells”, which are slightly different from CSCs and have recently been recognized to be crucial in the development of resistance to chemotherapy. However, the definitions and usage of these terms are currently unclear, and it is inconclusive whether these new cell populations are different cellular fractions from CSCs. In this section, we first compare in detail the papers in this field, which include not only organoids but also cell lines and PDX-based studies, and then organize the organoid-related papers according to the classification of cells along a timeline that we have conceived as a result of this comparison.

### 3.1 Cancer stem, slow-cycling, drug-tolerant persister, resistant or what?

Emerging evidence indicates that CSCs are a major cause of treatment resistance (Li et al., 2021). CSCs are a subset of tumor cells with self-renewal and differentiation potential that acquire resistance to anticancer drug therapy through phenotypic changes (Clevers and Nusse, 2012; Zhang et al., 2017). There are several clones of CSCs, which are thought to readily adapt in response to changes in the tumor microenvironment, radiation, and chemotherapy (Steinbichler et al., 2018). In contrast, a growing number of publications have recently described cell populations other than CSCs, highlighting the existence of cells specialized for drug resistance, called drug-tolerant persisters (DTPs), and very slow-growing cell populations, called dormant or slow-cycling cells. The identification of resistance-associated cells and their mechanisms has long been a subject of discussion (Borst, 2012; Mikubo et al., 2021) and is an inevitable topic when discussing anticancer drug resistance-related research using organoids.

Regarding DTPs and slow-cycling cells, in 2010, Sharma et al. (2010) showed by using cell lines that cancer recurrence may be caused by quiescent persister cells resulting from transient reversible resistance, besides the presence of rare cancer clones with drug resistance. Furthermore, it is suggested in cell line and PDX-based studies that these persister cells acquire hereditary drug resistance after a long latency period (Ramirez et al., 2016; Russo et al., 2019). Together, these findings raise the possibility that the persistence of so-called DTPs during the early stages of chemotherapy may be the initial step in the acquisition of resistance and relapse. The relation between these DTPs and stemness has also been investigated, and DTPs in glioblastoma have been reported to exhibit stem cell-like properties and slow cycling (Liau et al., 2017).

There is also much discussion about whether these cell populations are present before chemotherapy. Barcode sequencing analysis using non-small cell lung cancer cell lines has shown that particular subsets of cells present before treatment are more likely to acquire erlotinib resistance (Bhang et al., 2015). In addition, single-cell transcriptomes of aromatase inhibitor-resistant cell lines have shown that particular plastic cells in tumor tissue are more likely to attain resistance (Hong et al., 2019). Single-cell analysis of PDXs and cell lines has shown that the phenotype of cells can predict drug efficacy to some extent and can also predict the development of resistance (Georgopoulou et al., 2021). Furthermore, single-cell analysis of samples derived from patients receiving neoadjuvant chemotherapy (NAC) has shown that resistant cells exist before treatment and are adaptively selected by the NAC, reprogramming their transcriptional profiles in response to the NAC (Kim et al., 2018). Ohta et al. (2022) have shown that Lgr5+p27 + cells among Lgr5+ CSCs are a dormant subclone involved in tumor repopulation after chemotherapy using organoids and their xenografts in vivo imaging. As described above, the presence of DTPs or their progenitor cells before treatment has been reported in many cases, which could be a result of DTPs and CSCs having some commonality.

In contrast, Rehman et al. (2021) performed DNA barcode sequencing analysis using colorectal cancer-derived PDXs and showed not only that chemotherapy does not enrich certain cancer clones but also that all cancer cells have an equal potential to become DTPs. DNA barcode sequencing studies using organoids also suggested that there is no presumptive pre-existing cell population or rare clone with drug-resistant features before treatment (Dhimolea et al., 2021). However, the identification of a persister population distinct from CSCs has been reported (Echeverria et al., 2019), as has the loss of the LGR5+ CSC population in the persister state (Solé et al., 2022). Considering these conflicting results, it is difficult to conclude at this point whether CSCs and DTPs share a commonality or whether clonal selection/expansion occurs when chemotherapy is administered.

Further, the existence of “cycling persister cells” has been reported as a new subpopulation identified in recent years (Oren et al., 2021). Although most cells that survive after chemotherapy are slow-cycling, a very small population of persister cells with rapid proliferative potential has been identified. This subpopulation has also been shown to be present before treatment. This report raises the possibility that the cells involved in the progression of disease during chemotherapy may be completely different from those involved in recurrence after a certain period. If confirmed, this could bring a new dimension to current anticancer therapies. Therefore, there is still much to discuss regarding the cell populations that contribute to anticancer drug resistance and recurrence, and further detailed studies are still urgently needed.

**FIGURE 4:**
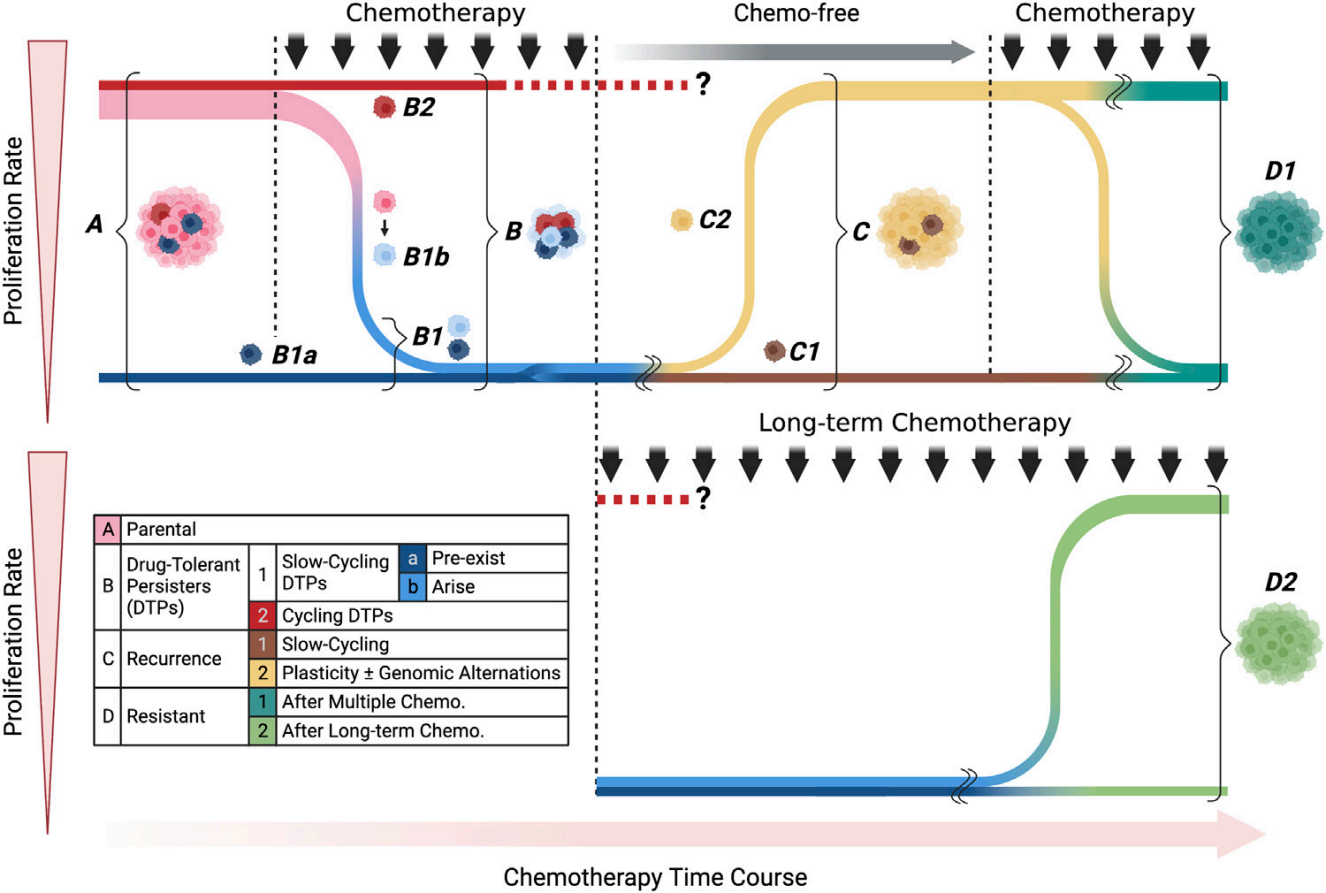
Classification of treatment-survived cells by proliferation rate and chemotherapy time course. Each color corresponds to the cell classifications shown in the included table. We divided the cell populations into four main categories: parental, drug-tolerant persisters (DTPs), recurrence, and resistant. In DTPs, we showed slow-cycling and cycling subcategories, pre-exist and arise populations in detail. Recurrence populations include slow-cycling and cycling populations, which could arise due to cell plasticity and/or genomic alternations. The resistant population was categorized according to the timeline of treatment: multiple or long-term chemotherapies.

Considering the recent findings, distinct biological characteristics are likely to be present among treatment-surviving cells: the cells that survive the initial phase of chemotherapy, the cells that show proliferative potential under exposure to anticancer agents, and the cells with treatment resistance that arise after chemotherapy. It is also unclear whether they were triggered by chemotherapy. These issues may be closely related to the time course of chemotherapy, suggesting the need for an organizing approach that reflects the treatment time course. In reviewing the articles presented so far from this perspective, they all commonly use terms such as CSCs, DTPs, and slow cycling, but the position of the cell populations to which each of these terms refers in the treatment time course is highly disparate. Furthermore, clear definitions of these terms have not been established, which is part of the barrier to better understanding in this field. We have summarized the cell populations analyzed in the previous publications related to anticancer drug resistance by categorizing them according to their treatment time course (Figure 4; Table 1).

**TABLE 1:** Classification of analysis targets in publications on treatment-survived or regrown cells.

Author	Year	Model	Drug mainly used	Analysis target	Key words
Sharma	2010	Cell line, Mouse model	EGFRi, RAFi	B1	drug-tolerant persister, quiescent
Bhang	2015	Cell line	EGFRi, ALK inhibitor, ABL1i	D2	resistant clone
Ramirez	2016	Cell line	EGFRi	D2	drug-tolerant persisters, persister-derived drug-resistant
Liau	2017	Cell line	BCR-ABL TKI	A, B, C	drug-tolerant persisters, slow cycling, quiescent, resistance
Kim	2018	Patient tissue	Anthracycline, Taxane, VEGFi	A, B, D2	therapy resistance, persistence
Echeverria	2019	PDX	Anthracycline, Alkylating agent	A, B, C	drug-tolerant, chemoresistance
Hong	2019	Cell line, Patient tissue	Aromatase inhibitor	A, D2	resistance, pre-adapted cells
Russo	2019	Cell line, PDX	anti-EGFR antibody, BRAFi	A, B1, D2	drug-tolerant (persister), drug-resistant, permanently resistant
Engel	2020	PDO	5-FU	B	drug-resistance
Ganesh	2020	PDO, PDX, Patient tissue	CPT-11	A, B, D2	chemoresistance, quiescence
Ukai	2020	PDO, PDX	5-FU	A, D2	drug-resistance
Zhang	2020	Cell line, PDO, PDX	Antiandrogen	A, B, D2	resistance
Dhimolea	2021	Cell line-derived Organoid, PDO, PDX	Anthracycline, Taxane	B1, C	drug persistance, embryonic diapause, diapause-like, proliferative quiescence
Georgopoulou	2021	Cell line, PDX	high-throughput drug-response experiments	A	resistance
Harada	2021	PDO	L-OHP	A, D2	drug-resistance
Oren	2021	Cell line, PDX	EGFR inhibitor	B1, B2	cycling cancer persister
Raghavan	2021	PDO, Patient tissue	Gemcitabine, Taxane, CPT-11(SN-38)	A, B	drug response
Rehman	2021	PDX	CPT-11, FOLFIRI	B1, B, C, D2	drug-tolerant persisters, slow cycling, diapause-like, irreversibly resistant
Takashima	2021	PDO	L-OHP	A, D2	drug-resistance
Álvarez-Varela	2022	PDO, Mouse model	5-FU, L-OHP, CPT-11(SN-38)	A, B1a, B, C	drug-tolerant persister, slow proliferative, chemoresistant, revival/fetal-like
Laoukili	2022	PDO	L-OHP	A	drug-resistance
Nicolas	2022	PDO, Mouse model	5-FU, irradiation	A, B, C	resistance
Ohta	2022	PDO, PDX	CPT-11, 5-FU, L-OHP	B1a, C2, C	dormant, persistent, slow-cycling, quiescent, reversible drug-tolerant state
Solé	2022	PDO	5-FU, CPT-11	B1, C	fetal signature, non-senescent, persistent quiescent-like

Analysis target refers to Figure 4. PDO, patient-derived organoid; PDX, patient-derived xenograft.

As shown in the table, although there was a slight variation in the combination of keywords and timeline cell types that appeared in the papers, surviving cells in the early stages of treatment were generally mentioned as having slow proliferation (Table 1). Note, that only Oren et al. (2021) reported cycling persister cells showing proliferation in the early stage of treatment. Other studies focused on cells during a complex treatment time course, such as relapse when treatment is interrupted, resistance to re-treatment, and resistance that occurs during long-term treatment. By focusing on the time axis, it becomes clear that the analysis targets may be completely different even if the articles seemingly use the same keywords (Figure 4; Table 1). If we can clarify the functional and molecular biological differences among these cells, this may be a new approach to overcoming the problem of anticancer drug resistance we are currently facing.

We next present four major categories of research papers in the field of anticancer drug resistance using organoids, considering the experimental methods and the time points of the chemotherapy to be analyzed.

### 3.2 Research on mechanisms of anticancer drug resistance using organoids

#### 3.2.1 Direct comparison of pre- and post-chemotherapy specimens and organoids

By comparing surgical specimens collected before and after chemotherapy and organoids established from these tissues, we can capture the actual chemotherapy-induced changes that occurred *in vivo*. The L1 cell adhesion molecule (L1CAM), which is significantly upregulated in tissues after NAC, has been shown to increase irinotecan sensitivity in organoid-based knockdown assays (Ganesh et al., 2020). Laoukili et al. (2022) compared susceptibility testing of organoids established from peritoneal metastasis of colorectal cancer with organoids derived from primary colorectal tumors and showed that the former were more resistant to oxaliplatin, and identified glutamate-cysteine ligase as a contributing factor. Although these methods have the advantage of directly assessing cells that have remained and expanded during actual chemotherapy, it is difficult to focus on the detailed treatment time course and to capture the changes and plasticity that may have occurred in the cells as a result of the treatment.

#### 3.2.2 Studies in which cells survived after treatment of organoids with anticancer drugs

Cells that remain just after treatment are often referred to as DTPs and are likely to be involved in the development of treatment resistance and recurrence. Understanding the characteristics of these cells is a prerequisite to understanding the early stages of treatment failure. Engel et al. (2020) reported that clusterin, a marker of the revival stem cell population, is significantly enriched in organoids after 5-fluorouracil (5-FU) treatment, and its expression correlates with the resistance level to 5-FU. Dhimolea et al. (2021) exposed organoids to anticancer drugs over a short to medium time course and identified residual cells as treatment-persistent organoids. Furthermore, they showed that transcriptome changes in these persistent cells are consistent with those observed in samples obtained before and after NAC. In addition, inhibition of Myc or Brd4, a transcriptional co-activator of Myc, in cancer cells attenuates the cytotoxicity of anticancer drugs through an adaptation to dormant diapause-like conditions. Ohta et al. (2022) showed by *in vivo* imaging of subcutaneously implanted organoids in mice that Lgr5 + p27 + cells remain after chemotherapy, further indicating that they are also involved in tumor regrowth *via* the COL17A1 and FAK-YAP pathways. Solé et al. (2022) treated organoids with IC20 and IC30 concentrations of 5-FU + Iri and performed RNA-seq, which showed that resistance is associated with WTp53, and coincidentally found loss of the LGR5+ CSC population in the persister state. Further investigations are needed to delve deeper into the relation between DTP cells and CSCs and how their populations change before and after chemotherapy.

#### 3.2.3 Analyzing repopulated cells after anticancer drug therapy in organoids

In the field of drug resistance research, a method of establishing resistant cell lines has generally been used in which cell lines are exposed to anticancer drugs for months to years. This method targets cells at the next stage of DTPs or the final stage of resistance acquisition. 5-FU resistance in gastric cancer organoids has been successfully established, with comprehensive genetic analysis identifying KH RNA Binding Domain Containing, Signal Transduction Associated 3 (KHDRBS3) as a novel gene responsible for 5-FU-resistance (Ukai et al., 2020). The establishment of oxaliplatin-resistant organoids has also been studied, and an association between the regulation of Schlafen11 (SLFN11) and myoferlin (MYOF) expression with drug sensitivity has been reported (Harada et al., 2021; Takashima et al., 2021). What these studies reveal is a feature associated with complete acquired resistance, which is the ability to proliferate even under exposure to anticancer drugs, and this should be considered separately from the DTPs.

#### 3.2.4 Studies using organoids targeting resistance caused by the association with tumor microenvironment, cancer-associated fibroblasts (CAFs), and immune cells

One of the characteristics of organoid cultures is that they can be easily co-cultured with CAFs and immune cells. Moreover, the organoid culture medium can be easily modified to reproduce alternations and diversity in the tumor microenvironment by adding or deleting niche factors. Zhang et al. (2020) used organoids to show that neuregulin 1 (NRG1) secreted from CAFs is associated with anti-androgen resistance in prostate cancer. Inflammatory CAFs have also been shown to be involved in radiotherapy resistance in rectal cancer using organoids and orthotopic transplantation models in mice (Nicolas et al., 2022). Besides, the detailed analysis by Raghavan and others (2021) of the association between cell state and microenvironment in pancreatic cancer using bulk RNA-seq and scRNA-seq of organoids indicates that differences in the microenvironment-driven cell state can significantly alter anticancer drug sensitivity. Álvarez-Varela et al. (2022) identified Mex3a, a potential DTP colorectal cancer cell state marker due to the failure of LGR5+ stem-like cells to adapt to the niche, by culturing organoids with additional TGF-β or without EGF. Furthermore, they showed that after chemotherapy, Mex3a + cells clone and regenerate lesions. Thus, by using organoids, it is possible to examine not only the characteristics of cancer cells but also the interactions of cancer cells and their surrounding environment, enabling the capture of disease closer to that in the *in vivo* state. The functions and roles of non-cancer cells are expected to attract more attention in the near future as one possible solution needed to break through the limitations of current precision medical treatment. Furthermore, demonstrating this in combination with the treatment time course will lead to a more accurate understanding of the disease.

## 4 Association of organoid drug sensitivity with clinical outcomes for precision medicine

Unresectable or relapsed patients are often treated with chemotherapy, but as no chemotherapy is equally effective for all patients, selection of the optimal treatment for individual patients is required. Although the advance of personalized medicine has been achieved to some extent with the advent of molecular targeted therapies, the efficacy of such therapies often varies even within the specific patient group for which they are indicated. Predicting efficacy before chemotherapy is still challenging, and the development of such methods will be crucial for the advancement of personalized medicine. In this context, cancer organoids are superior in the following points: a high success rate of establishment, ability to reproduce not only pathological features but also intra-tumor heterogeneity, and easy *in vitro* investigation of drug response of cancer cells. In this section, we focus on studies examining the correlation between actual clinical outcomes and experiments using organoids.

Yan et al. (2018) showed that the results of a drug sensitivity assay using gastric cancer organoids correlated with the actual therapeutic efficacy of cisplatin- and 5-FU-based chemotherapy. In a report on bladder cancer, the results of a drug screening using organoids were validated in a mouse xenograft model showing consistent results (Lee et al., 2018). Sachs et al. (2018) generated human-derived breast cancer organoids and showed that the results of their *in vitro* drug sensitivity examinations were in line with those of *in vivo* experiments using xenografts and the clinical outcome. Organoids established from olaparib-resistant ovarian tumors were also highly resistant to olaparib during *in vitro* drug susceptibility assays. It was also noted that the clinical outcome was largely consistent with the results of susceptibility testing to other anticancer drugs (Hill et al., 2018). In the esophageal cancer organoids established by Li et al. (2018), the results of *in vitro* drug sensitivity assays also reflected the results of NAC and the effectiveness of adjuvant treatment. Cisplatin resistance was also shown to be reproduced by *in vitro* assays in organoids established from cisplatin-resistant mesothelioma (Mazzocchi et al., 2018). Investigations using metastatic gastrointestinal cancer-derived organoids have shown that drug sensitivity tests for organoids recapitulate the therapeutic effects observed in clinical practice with very high accuracy. Generation of xenografts from organoids has enabled testing of the antitumor effects of regorafenib, an anti-angiogenic drug, and the prognosis of treated mice has been reported to correlate with clinical outcomes (Vlachogiannis et al., 2018). Treatment-naive rectal cancer organoid biobank studies have illustrated that the results of organoid-based chemoradiotherapy trials are consistent with actual clinical outcomes (Yao et al., 2020). Ovarian cancer organoids established by de Witte et al. (2020) recapitulated the patient response to carboplatin and paclitaxel treatment. Jiang et al., 2020) reviewed computed tomography of colon, rectal, and liver tumors in three patients to confirm the results of screening for organoids and found computed tomography and screening results to be consistent in cases of both successful and unsuccessful treatment. Grossman et al. (2021) examined the association of clinical outcomes with screening results of 12 pancreatic cancer organoids and seven anticancer agents. Although multiple anticancer agents were used in clinical practice, they reported that treatment responded when at least one organoid-sensitive agent was included in the regimen. Chen et al. (2021) examined the association between drug sensitivity testing and treatment regimens for breast cancer organoids. They reported that 71% of patients who received one or more drugs classified as sensitive achieved stable disease or partial response, whereas 93% of patients who received only the non-sensitive drugs experienced progressive disease. They also documented preclinical results with these organoids with an area under the curve of 80.1%. Based on these reports, it is widely considered that, in general, the results of drug efficacy studies with organoids are consistent with clinical outcomes, with one partial exception.

### 4.1 Indication of the need to optimize experimental conditions, including microenvironment

Drug sensitivity of organoids is shown to be consistent with clinical outcomes in most settings. However, studies of multiple metastatic colorectal cancers show discrepancies in their results. Pasch et al. (2019) established metastatic colorectal cancer organoids to examine the correlation between drug sensitivity test results and clinical outcomes. Results confirmed that the efficacy of the 5-FU and oxaliplatin combination seen in organoids is consistent with actual clinical reductions in tumor size and tumor markers. Meanwhile, Ooft et al. (2019) studied clinical outcomes and results from drug sensitivity assays using metastatic colorectal cancer organoids, but they reported that the responses to irinotecan alone and 5-FU + irinotecan were consistent for both agents but not for the combination of 5-FU and oxaliplatin. Interestingly, the two studies draw different conclusions concerning whether organoids can predict the outcome of combination therapy with 5-FU and oxaliplatin. There are many differences in the experimental methods used in the two papers, which may be the reason for the different conclusions; Ooft et al. (2019) used Geltrex but Pasch et al. (2019) used Matrigel, and the medium components were also largely different, with the Pasch et al. medium not containing B27, N-acetylcysteine, gastrin, or N2 supplement. However, it is difficult at this stage to evaluate and optimize the properties of the culture medium, and it is problematic that the current organoid technology cannot fully reproduce the varied microenvironment for each patient within the organoid culture because the stroma and immune system are not present in the culture.

Zitvogel et al. (2008) pointed out that such differences in the tumor microenvironment may influence therapeutic response in various ways. For example, Lin et al. (2019) reported that the EGF/ATXN2L axis promotes oxaliplatin resistance, from which we can predict that the amount of EGF added to the organoid medium may alter oxaliplatin sensitivity. This may explain why *in vitro* and clinical treatment results do not always match. Laoukili et al. (2022) also reported that N-acetylcysteine, a substance frequently added to organoid media, affects oxaliplatin sensitivity of colorectal peritoneal metastases-derived organoids. Therefore, a possible solution is to improve the medium composition, but considering the heterogeneity of CAFs, which mainly comprise the microenvironment (Ishii et al., 2016), they should be considered when determining the optimal medium composition for individual organs and patients. One possible example is to apply the functional classification of CAFs based on secreted factors such as HGF and FGF7, as shown by Hu et al. (2021). The addition of these factors to the organoid medium could optimize the medium composition. However, given the limited data available to date, further studies on the relation between organoid culture bias, medium composition, microenvironment, and sensitivity to anticancer drugs are needed in the future. Nevertheless, the number of cases included in these two publications is limited, and it is not possible to come to any conclusions based only on the results of these studies. The results imply that further molecular biological approaches are needed to evaluate the possibility of interactions between tumor cells and stromal cells, and to improve the reproducibility of the microenvironment constructed by these cells.

As described above, although many papers have shown a correlation between clinical information and the results of drug susceptibility examinations using organoids, the number of cases presented in each paper is still limited, and among these reports, some come to different conclusions. Other approaches to this challenge include examining associations with clinical outcomes in larger cohorts, and clinical trials should also be conducted extensively.

### 4.2 Organoids in clinical trials

Only a few clinical trials using organoids have been reported. In addition to the aforementioned report by Ooft et al. (2019), a prospective study in colorectal cancer patients with peritoneal metastasis reported that the treatment of two patients was modified based on the results of an organoid drug sensitivity assay, of which one patient had a successful response (Narasimhan et al., 2020). Wang et al. (2021) conducted a blinded study using stage 4 colorectal cancer-derived organoids, and the observed accuracy was 79.69%. A prospective clinical trial using metastatic colorectal cancer organoids was also conducted as an open-label, single-center, prospective, feasibility study (Ooft et al., 2021). Thirty-one organoids were successfully established from 54 of 61 patients, and 25 cultures were screened for agents, with 19 organoids reported to be sensitive to one or more agents. Among them, 3 were treated with vistusertib and 3 with capivasertib. However, despite the predicted response from the organoid studies, the patients did not show successful clinical responses to the recommended therapy. Possible solutions, they report, include optimization of the culture medium conditions and patient stringency.

According to a ClinicalTrials.gov search for ongoing clinical trials using organoid technology, there were 112 studies as of June 2022 with hits for the keyword “Organoid Cancer”. There were 25 lower gastrointestinal cancers, 19 breast cancers, 16 pancreatic cancers, 13 lung cancers, 9 upper gastrointestinal cancers, 7 ovarian cancers, as well as bile duct, liver, neuroendocrine tumor, head and neck cancer, renal cancer, and bladder cancer organoid-related trials also being registered. The conditions under which the organoids should be used for personalized medicine should be considered on the basis of these results. However, the need for optimization of culture media conditions is an important concern, as discussed in this review, and it is considered essential to improve the reproducibility of the tumor microenvironment, which differs from patient to patient. Toward the achievement of enhanced personalized medicine, detailed investigations based on molecular biological methods will become even more important, although the results of practicality verification through expanded clinical trials will also be important.

## 5 Drug screening (DS) methods and automated high-throughput assay for organoids

### 5.1 Variety in DS methods

Organoids can now be used to test various anticancer drugs in their preclinical stages, and the protocols are widely recognized (Driehuis et al., 2020). The platforms that appear in publications are very diverse. In most cases, Matrigel is used as the extracellular substrate, but other basement membrane extracts such as Geltrex are used in some papers (van de Wetering et al., 2015; Sachs et al., 2018; Ooft et al., 2019). When cells are plated, gels are often used undiluted as in general passages, although there are also reports of gel being diluted with a culture medium before experimentation (Ooft et al., 2019; Narasimhan et al., 2020; Wang et al., 2021). Other conditions include the type of cell separation reagent; the well size of the plates used; the period required for cell seeding, reagent addition, and measurement; the type of measurement reagent; and many others. These conditions affect the efficiency of organoid establishment and proliferation, possibly resulting in different results from one laboratory to another even in the same experiment. Standardized criteria are needed for future clinical applications.

**FIGURE 5:**
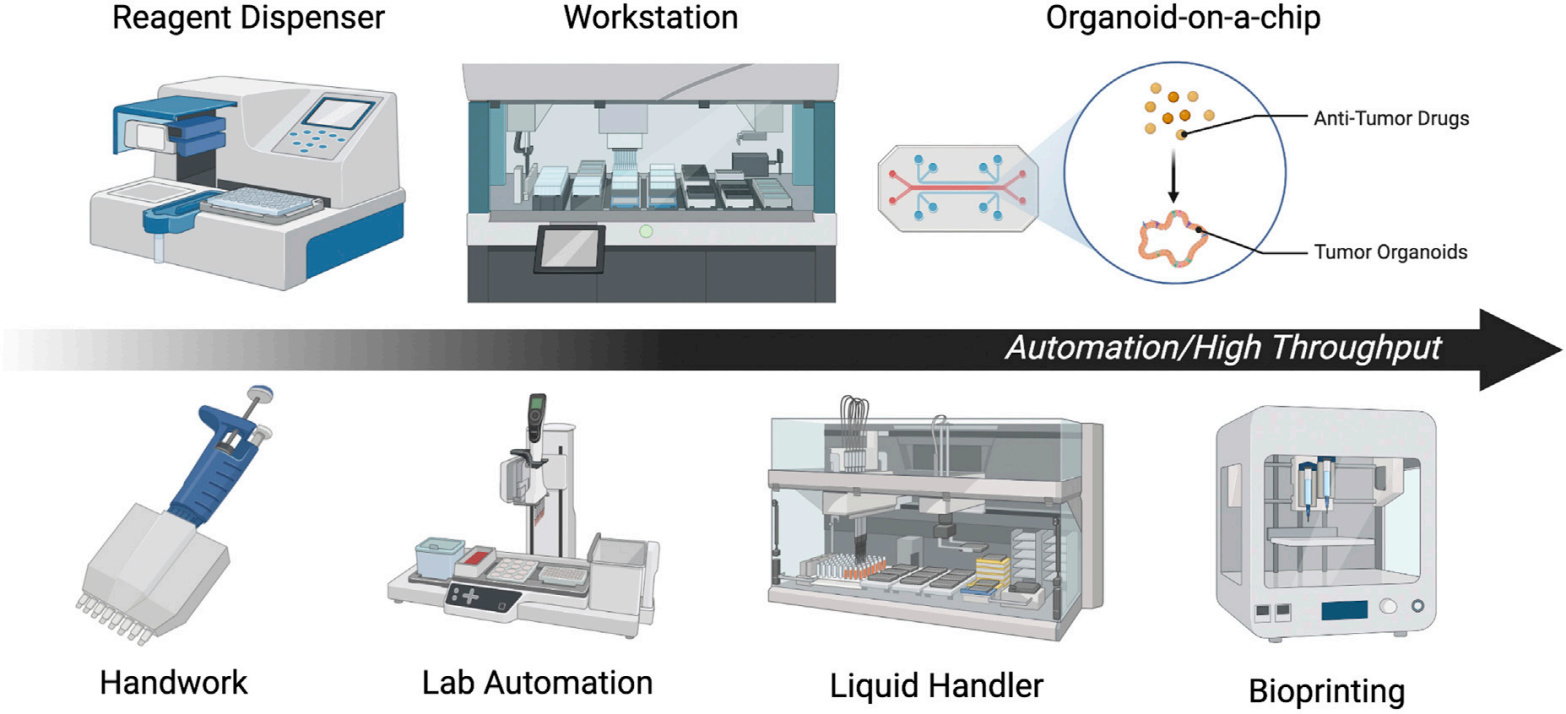
Summary of automation and high-throughput methods. Each illustration is a scheme of representative automation or high throughput methods.

To date, most researchers have performed DS assay manually, but in recent years, automated and high-throughput DS methods using organoids have been well developed, and their variations have been growing. The feasibility of applying organoids to personalized medicine is expected to be enhanced by the development of technologies as summarized below (Figure 5).

### 5.2 Automation and high throughput of DS

To perform stable screening with a larger number of cases and drugs, high-throughput and non-labor-intensive drug susceptibility testing methods are essential. It should also be noted that performing all organoid seeding manually when conducting DS can lead to significant batch-to-batch and well-to-well variances (Jiang et al., 2020). To reduce well-to-well variation during drug addition, the Thermo Multidrop Combi Reagent Dispenser or similar devices have been used (Yan et al., 2018), but there remains a need for total standardization. Automation and high-throughput drug sensitivity assays using organoids can be broadly divided into the cell plating, drug addition, and plate reading steps.

Brandenberg et al. (2020) developed a microengineered hydrogel membrane at the base of a conventional multi-well plate to simultaneously derive thousands of uniform organoids at predefined positions on the same focal plane. Jiang et al. (2020) have also successfully automated cell plating by mixing cell-suspended Matrigel and volatile cell-compatible oil (HFE7000) in microfluidics with a cooling system. A Matrigel droplet of equal size is formed in the cooled oil, which is then passed through a heated tube to gelatinize and form the complete droplet. The droplet is then placed in each well by a bioprinter and used for subsequent assay. These authors have shown that each droplet contains a constant number of cells, each of which retains the heterogeneity of the tumor. Matrigel is often formed in a dome shape, but the use of a “ring format” in which Matrigel is plated along the wall of each well has also been shown to be useful. Indeed, high-throughput drug sensitivity testing using this method has been shown to predict a carboplatin non-responder (Phan et al., 2019). For drug addition and plate reading, Schuster et al. (2020) developed a system that enables combinatorial and dynamic drug administration to hundreds of samples, resulting in a platform that analyzes organoid survival in real time. The platform consists of a 3D culture chamber, multiplexer fluid control system, customizable software, and a live cell time-lapse fluorescence microscope that can measure gene expression changes and survival rates over time for up to 20 samples.

Alternatively, there are several studies aiming at total automation of high-throughput DS by combining currently effective systems. Pauli et al. (2017) conducted conventional DS methods using lab automation, which refers to hardware and software, such as highly scalable plate handlers, that can enable safer, faster, and more accurate experiments than those performed manually. In addition, Brandenberg et al. (2020) successfully automated most of the steps from organoid culture to measurement by combining automated cell plating technology with a robotic liquid handling system. Further, Choo et al. (2021) combined a JanusG3 liquid handling robot, LiCONiCs STX220 high-throughput incubator, Tecan D300e drug printer, Sciclone ALH3000 robot, Cytation5 Cell Imaging Multi-Mode Reader, and other devices to successfully automate all steps of DS. In addition, a complex automated platform has been created that combines not only a liquid handler and droplet ejector but also a cell counter, robotic arm, incubator, cooling centrifuge, and multimode plate reader. This platform can be applied to culture systems, including organoids (Boussaad et al., 2021).

The combination of each of the latest technologies described so far will enable standardized DS to be performed in many laboratories. Furthermore, recently developed technologies such as organ-on-a-chip (Junaid and Hankemeier, 2021) could also be used for drug susceptibility testing of organoids, providing further potential for advancement.

## 6 Conclusion

The organoid model is one of the outstanding preclinical models, and this review summarizes the latest findings under the headings of morphological and molecular biological characteristics of cancer organoids, resistance mechanisms studied using organoids, the relation between organoid drug sensitivity assays and clinical outcomes, and automation and high throughput. In the process, critical issues related to chemotherapy resistance emerged. These include the need for clear definitions and characterization of which cells at which stage during the treatment time course are to be analyzed, optimization of media, and standardization of techniques. Overcoming chemotherapy resistance has long been a goal, and as a clear understanding of the mechanisms is the first step toward a solution, further research will be required to achieve this goal. We believe that the effective use of non-clinical models will directly affect the lives and quality of life of many patients.
