# Supplementary material for: Cancer organoid applications to investigate chemotherapy resistance
Source: Front Mol Biosci. 2022 Dec 13;9:1067207. doi: 10.3389/fmolb.2022.1067207 (PMC9792487; doi:10.3389/fmolb.2022.1067207)
Supplement: Supplementary file 1 [file DataSheet1.pdf]

Supplementary Table S1 List of media ingredients, related to Figure 3.

	Publication	Year	Basic Medium	EGF(ng/ml)	B27	N-acetylcysteine (mM)	A83-01(nM)	Glutamax(mM)	Noggin (ng/ml)	Y-27632(uM)
Gastric Cancer	Bartfeld et al.	2015	Advanced DMEM/F12	50	1x	1	2000	2	10%CM	10
	Yan et al.	2018	Advanced DMEM/F12	50	1x	1	2000	1x	10%CM	10
	Steele et al.	2019	Advanced DMEM/F12	50	1x	1	-	L-Glu.(Thermo) 1x	100	10
	Lo et al.	2021	Advanced DMEM/F12	50	1x	1	500	2	50%CM	10
	Togasaki et al.	2021	Advanced DMEM/F12	50	1x	1	500	2	100	10
Colorectal Cancer (CRC)	Sato et al.	2011	Advanced DMEM/F12	50	1x	1	500	2	100	10
	van de Wetering et al.	2015	Advanced DMEM/F12	50	1x	1.25	500	+	10%CM	10
	Engel et al.	2020	Advanced DMEM/F12	50	1x	1.25	500	+	10%CM	10
	Ganesh et al.	2020	Advanced DMEM/F12	50	1x	2	500	2 (Glutamine)	50	10
	Della Chiara et al.	2021	Advanced DMEM/F12	50	1x	1	500	2	100	-
	Solé et al.	2022	Advanced DMEM/F12	50	1x	1.25	500	-	100	10
	Wang et al.	2022	Advanced DMEM/F12	50	1x	-	500	2	100	10
Peritoneal Metastases CRC	Narasimhan et al.	2020	Advanced DMEM/F12	50	2x	1	500	1x	-	10
	Laoukili et al.	2022	Advanced DMEM/F12	+	1x	1.25	500	0.4	0.1	-

	Publication	P/S (U/ml)	Nicotinamide (mM)	HEPES (mM)	R-spondin (ug/ml)	GastrinI(nM)	Wnt-3a(ng/ml)	FGF-10 (ng/ml)	SB202190 (uM)	Primocin(ug/uL)	N2 supplement
Gastric Cancer	Bartfeld et al.	100	10	10	10%CM	1	50%CM	200	-	-	-
	Yan et al.	1x	-	1x	50%CM	1	50%CM	200	-	1	-
	Steele et al.	100	10	10	CM	1	CM	200	-	-	1x
	Lo et al.	50	10	1	50%CM	10	50%CM	200	10	-	-
	Togasaki et al.	100	-	10	1	10	25%CM (Afamin-Wnt3a)	50	-	-	-
Colorectal Cancer (CRC)	Sato et al.	100	10	-	1	10	100	-	10	-	1x
	van de Wetering et al.	+	10	10	20%	10	-	-	3	100	-
	Engel et al.	-	10	10	20%CM	10	-	-	-	100	-
	Ganesh et al.	-	10	10	1	10	100	-	10	100	1x
	Della Chiara et al.	+	10	10	-	10	-	-	10	-	1x
	Solé et al.	100	10	-	0.1	10	-	-	3	100	1x
	Wang et al.	100	4	-	0.5	10	100	10	5	+	-
Peritoneal Metastases CRC	Narasimhan et al.	-	-	10	-	1	-	-	5	-	-
	Laoukili et al.	50	10	10	0.1	10	-	-	10	-	-

	Publication	Gentamicin	Amphotericin B	EDTA(mM)	Dnase(U/ml)	SB431542 (uM)	FGF basic (ng/ml)	PGE2(uM)	Kanamycin	FBS	Normocin(ug/ml)	Antibiotic-Antimycotic
Gastric Cancer	Bartfeld et al.	-	-	-	-	-	-	-	-	-	-	-
	Yan et al.	-	-	-	-	-	-	-	-	-	-	-
	Steele et al.	1x	1x	-	-	-	-	-	1x	-	-	-
	Lo et al.	-	-	-	-	-	-	-	-	5%	-	-
	Togasaki et al.	-	-	-	-	-	-	-	-	-	100	-
Colorectal Cancer (CRC)	Sato et al.	-	-	2	2000	-	-	-	-	-	-	-
	van de Wetering et al.	-	-	-	-	-	-	0.01	-	-	-	-
	Engel et al.	-	-	-	-	-	-	-	-	-	-	-
	Ganesh et al.	-	-	-	-	-	-	1	-	-	-	-
	Della Chiara et al.	-	-	-	-	-	-	-	-	-	-	-
	Solé et al.	-	-	-	-	-	-	0.01	-	-	-	-
	Wang et al.	-	-	-	-	-	10	0.1	-	-	-	-
Peritoneal Metastases CRC	Narasimhan et al.	10mg/L	-	-	-	10	-	-	-	-	-	1x
	Laoukili et al.	-	-	-	-	-	-	10	-	-	-	-
